# Improving predictability of IgE-high type 2 chronic sinusitis with nasal polyps in the biologic era

**DOI:** 10.1186/s40463-022-00580-y

**Published:** 2022-05-23

**Authors:** Austin Heffernan, Jobanjit Phulka, Andrew Thamboo

**Affiliations:** 1 Division of Otolaryngology – Head and Neck Surgery, Department of Surgery, University of British Columbia, 2775 Laurel Street, 4th Floor, Vancouver, BC V5Z 1M9 Canada; 2 Division of Otolaryngology – Head and Neck Surgery, Department of Surgery, St. Paul’s Hospital, Vancouver, BC Canada

**Keywords:** Chronic Rhinosinusitis, Immunoglobulin-E, Biomarkers, Endotype, Monoclonal antibodies, Biologics, Therapeutics

## Abstract

**Background:**

Chronic rhinosinusitis (CRS) is an inflammatory disease that may require biological therapy. Omalizumab is an anti-IgE biologic that was recently approved by the FDA and Health Canada for use in severe CRS with nasal polyps (CRSwNP) recalcitrant to intranasal corticosteroids. Dosing is based on weight and pre-treatment serum IgE, with elevated levels of the latter being an indication for biologic treatment according to EPOS and EUFOREA guidelines. The goal of this study was to identify variables that predict IgE-high type 2 inflammation and serve as indicators for biologic treatment in CRS.

**Methods:**

Patients ≥ 19 yo diagnosed with CRS undergoing functional endoscopic sinus surgery were included retrospectively. Demographics, past medical history, preoperative blood work, Lund-Mackay (LM), Lund Kennedy (LK), and SNOT-22 scores were extracted. Descriptive statistics and binary logistic regression analyses were conducted. Model superiority was based on Nagelkerke R2 scores and receiver operating characteristic curves**.**

**Results:**

Sixty-five patients, average age 49.96 ± 13.59 years, were included. Sixty-one binary logistic regression models for elevated serum IgE were created. Among the top 3 models, the best model had sensitivity, specificity, positive predictive value and negative predictive values of 82.1, 69.2, 80.0, and 72.0. All performance measures except sensitivity exceeded the Canadian Biologics Guideline model. Serum eosinophils ≥ 300 cell/uL, CRSwNP and LM ≥ 17 increased the odds of elevated IgE.

**Conclusions:**

IgE-high type-2 inflammation can be predicted by a model that includes eosinophil ≥ 300 cell/uL, CRSwNP, LM ≥ 17, asthma diagnosis and SNOT-22 ≥ 40. Patients meeting these parameters have a high pretest probability for elevated IgE and would benefit from IgE serology to determine qualification for omalizumab. This could reduce unwarranted IgE serology in patients with CRSwNP but also target a patient population for further workup that will lead to optimization of resource allocation and improve healthcare equity in rural and remote areas within Canada.

## Background

Chronic rhinosinusitis (CRS) is an upper airway inflammatory disease of the sinuses and nasal cavity that affects 5.2% of the Canadian population [1]. This disease carries a significant health burden as patients suffer from rhinologic and extra-rhinologic symptoms. The former includes nasal congestion, purulent drainage, facial pain and anosmia, while the latter includes poor sleep, cognitive dysfunction, poor productivity, depression and anxiety [2, 3]. Based on these symptoms, it is understandable that in 2007, CRS consumed $8.6 billion in healthcare resources in the US [4]. These resources may have reduced the mortality risk of patients with CRS to that of the general population [5]. However, CRS is commonly classified by phenotype into CRS with nasal polyps (CRSwNP) or CRS without nasal polyps (CRSsNP), and the former has a 1.38 fold greater risk of mortality than the latter [5]. This stresses the importance of optimizing CRSwNP management.

CRS management utilizes pharmaceutical and surgical interventions to reduce symptoms and complications [4]. A subset of patients have symptoms that are refractory to treatment. This could be due to an oversimplified subclassification of CRS into CRSwNP and CRSsNP which has been shown to not accurately predict treatment outcomes [6]. In response, new classification systems have emerged. A now commonly used system categorizes patients by inflammatory endotype into non-type 2 (ie. non-eosinophilic) or type 2 inflammation (ie. eosinophilic). This is similar to asthma’s classification into type 2 high and type 2 low endotypes [7]. Having a similar endotype classification system makes it no surprise that biologic therapies have been approved for use in both type 2 asthma and type 2 CRSwNP [7–9].

These biological therapies seek to control the disease by mitigating the type 2 inflammatory response by targeting its respective cytokines and antibodies. Currently, biologic treatments have been and are being approved for the management of CRSwNP, which is phenotypically type 2 disease [10]. These include dupilumab (anti-interleukin (IL)4 and anti-IL13), omalizumab (anti-Immunoglobulin(Ig)E) and mepolizumab (anti-IL5) which target different aspects of the type 2 inflammatory cascade [9, 11, 12]. Omalizumab was recently approved by Health Canada in Sept 2021 as an add-on maintenance treatment with intranasal corticosteroids in adults with CRSwNP [13]. Its efficacy in CRSwNP has been confirmed by two randomized phase 3 trials, POLYP1 and POLYP2 [14]. A meta-analysis by Agache 2021 demonstrated that dupilumab, omalizumab and mepolizumab improved patients sense of smell and quality of life, and reduced the need for surgery [15].

The cost-effectiveness of these biologics is important to consider, especially in Canada’s single payee healthcare system. The annual cost of dupilumab is $25,909 CAD for year one and $24,949 CAD for subsequent years [16]. Similarly, the annual cost for mepolizumab is $25,269 CAD [16]. In contrast, the annual cost of omalizumab ranges from $3,564 CAD to $66,061 CAD based on dosage, which is dictated by baseline serum IgE levels and patient weight (kg) [14, 16]. Noteworthy, in the POLYP1 and POLYP 2 phase III trials the majority (~ 90%) of patients were on an every 4 week dosing schedule with a weighted average annual cost of approximately $19,000 to $20,000 (S. Parent (Novartis), personal communication, May 3, 2022) [14, 16]. It should be noted that these annual costs are based on list prices, which may not be the price paid by the payer due to confidential agreements between manufacturer and payer (G. Khaira (Sanofi), personal communication, May 3, 2022). Despite this, a cost-effectiveness analysis of biologics for CRS based on use patterns and outcomes in Canada is lacking. This explains why the systematic review by Agache 2021 failed to label one biologic to be superior when considering the balance of cost-effectiveness, benefits and adverse events data [15]. Furthermore, in the era of personalized medicine, it is important to keep medical options open as certain pharmacological agents may prove to be more beneficial for certain patients.

A baseline IgE level is an essential tool for dosing omalizumab. This may be a detriment to Canadian rural and remote hospitals which have access to basic blood work (complete blood count with differential, chemistry, toxicology) but not IgE serology. The latter requires blood samples to be sent to the laboratory at the nearest tertiary centre. This distance can be considerable in Canada due to its population density of 3.9 people per square kilometer with 66% of the population and most tertiary centres being located within 100 km of the United States border [17]. Shipping blood samples from beyond this distance can introduce cost and logistic barriers that could hinder omalizumab prescribing and reduce healthcare equity in rural and remote Canada. To mitigate this issue, we have investigated the use of common preoperative clinical, imaging and laboratory variables to predict elevated serum IgE levels and subsequent candidacy for confirmatory IgE serology. These variables should be readily available as the Canadian Rhinology Working Group indicates that patients should only be considered for biologic therapy if they are recalcitrant to sinus surgery and medical therapy [18].

## Methods

### Study design

This study was a retrospective chart review. Patients diagnosed with CRSwNP or CRSsNP based on clinical symptoms and endoscopic or radiographic evidence of inflammation were identified between August 1^st^, 2017 and December 31^st^, 2019. Patient data were collected and stored on University of British Columbia (UBC) Research Electronic Data Capture (REDCap) [19]. Ethics was obtained from the UBC Research and Ethics Board.

### Study population

All patients of the senior author who were ≥ 19 years of age, with a confirmed diagnosis of CRSwNP or CRSsNP who underwent functional endoscopic sinus surgery (FESS) and attended clinical visits at St. Paul’s Sinus Center between August 1st, 2017 and December 31st, 2019 were included.

### Data collection

Data were extracted from patients who met inclusion criteria. This data included sex, date of birth, date of surgery, and CRSwNP or CRSsNP. Patients with CRSsNP were included to assure validity of results and to determine if CRSsNP predicts IgE high type 2 inflammation [20, 21]. Assuring validity of results requires there to be little to no multicollinearity among independent variables. Therefore, including CRSsNP patients enabled CRS phenotype dichotomization and subsequent assessment of multicollinearity between all independent variables. Interestingly, recent studies from the United States and Europe have shown that a large proportion of patients with CRSsNP have a type 2 inflammatory endotype, but to a lesser extent than CRSwNP [20, 22–24]. This proportion in CRSsNP tends to fluctuate with geography. Therefore, we also included the CRSsNP patient population to determine if CRSsNP in Vancouver BC helped predict IgE high type 2 inflammation. Additional preoperative clinical parameters collected included preoperative Lund-MacKay (LM) CT staging score, Lund Kennedy (LK) endoscopic score and Sino-nasal Outcome Test-22 (SNOT-22) questionnaire score. Preoperative blood work including blood eosinophils and IgE levels was also collected.

### Statistical analysis

Descriptive statistics and a binary logistic regression were completed using the IBM SPSS® software platform. Descriptive statistics were first performed on age (mean, standard deviation) and sex (median, standard error) data.

Sophisticated variable selection approaches (ex. stepwise selection) were not required in this study due to the small number of predictors (n = 6) being studied. Variables used for statistical analysis were chosen based on statistical validity and clinical relevance. The former involved calculating the Pearson correlation coefficients (r) and variance inflation factor (VIF) for all studied variables. This is important because including highly correlated (r ≥ 0.7) variables in the binary logistic regression model can lead to inaccurate parameter estimates which reduces the validity of parameter interpretations. Similarly, VIF values need to be calculated to ensure there is minimal to no multicollinearity (VIF < 5) among independent variables to also improve the validity of results. IgE was selected as the response variable because it is the clinical variable used in prescribing omalizumab. Categorical predictors were set as CRSwNP status, serum eosinophilia, SNOT-22, asthma diagnosis and preoperative LK and LM scores prior to checking assumptions. These were chosen due to their roles as current and potential indicators for biological treatment for CRSwNP. According to the 2020 European Position Paper on Rhinosinusitis and Nasal Polyps (EPOS) and Canadian guidelines, CRSwNP is a requirement for biological treatment [25]. SNOT-22 and elevated serum eosinophils are quality of life and type 2 inflammation indicators respectively that are part of the criteria for biological treatment stipulated by 2020 EPOS and Canadian guidelines [18, 25]. Endoscopic scores such as LK was chosen due to it being used as an indicator for biological treatment by the Canadian and European forum for research and education (EUFOREA) CRS guidelines [18, 26]. However, LM score is currently not a required indicator for biological treatment and was included due to its potential to become an indicator.

These response variables were dichotomized in order for a binary logistic regression analysis to be performed. Predictor variables were also dichotomized because the histograms of their individual scores were skewed or non-normal. These cut-off values for dichotomization of IgE (≥ 250 ug/L), SNOT-22 (≥ 40) and eosinophilia (≥ 300 cells/uL), were based on biologics guidelines from EPOS and EUFOREA [25–27]. The cut-off values for the modified LK score and LM score were set at ≥ 4 and ≥ 17 respectively based on current CRS literature on mepolizumab, dupilumab and benralizumab biologics [11, 28, 29]. Furthermore, the response variable used in the model was coded as serum eosinophil levels ≥ 300 cells/uL and predictor variables were coded as CRSwNP (yes or no), asthma (yes or no), IgE levels ≥ 250 ug/L, SNOT-22 score ≥ 40, LK score ≥ 4 and LM score ≥ 17.

Best subsets selection was used to determine the best model for IgE high CRS. Model superiority was decided based on Nagelkerke R^2^ and receiver operator characteristic (ROC) curve area under the curve (AUC) values. Nagelkerke R^2^ was used to determine the proportion of variance in the outcome that is successfully explained by the model (ie. goodness of fit). Values closer to 1 indicated a higher quality model. The top 10 models were chosen based on higher Nagelkerke R^2^ values. Following this, the top 3 models were chosen based on their respective AUC values generated by the ROC curve. One additional model was included to represent current Canadian Biologic Guidelines and act as a relevant comparator for the best model in this study. The individual contribution of each variable to the best model is described by their respective odds ratio, confidence interval and Wald test significance value. Additional performance measures for each model are presented, including specificity, sensitivity, positive predictive value (PPV) and negative predictive value (NPV). Data output interpretation was completed through consultation with the University of British Columbia Department of Statistics and binary logistic regression guidance from the East Carolina University Department of Psychology [30].

## Results

A total of 65 patients were included in this retrospective review, which surpassed the one variable per ten participants rule for logistic regression analyses [31]. Thirty-five patients were diagnosed with CRSwNP and 30 patients were diagnosed with CRSsNP. The demographics of this sample population, including patient comorbidities associated with CRS, can be found in Table 1 [32].

**Table 1:** Demographics of the sample population

Demographic variable	CRSsNP	CRSwNP
Sample size (n)	30	35
Age (mean ± SD)	54.70 ± 12.19	47.51 ± 14.17
Sex (F:M)	16:14	17:18
Previous sinus surgery (n)	10	9
Previous septum surgery (n)	3	3
Allergy present (n)	12	20
*Comorbid conditions* (n)		
COPD	1	1
Bronchiectasis	0	2
Asthma	12	14
ASA sensitivity	0	3
Allergic rhinitis	0	1
GERD	1	3
Liver disease	1	0
Inflammatory/autoimmune disease	4	1
Psychiatric/neurological disease	2	3

ASA, Acetylsalicylic acid; COPD, Chronic obstructive pulmonary disease; CRSsNP, Chronic rhinosinusitis without nasal polyps; CRSwNP, Chronic rhinosinusitis with nasal polyps; F, female; GERD, Gastroesophageal reflux disease; M, Male; N, number of people; SD, Standard deviation

**Fig. 1:**
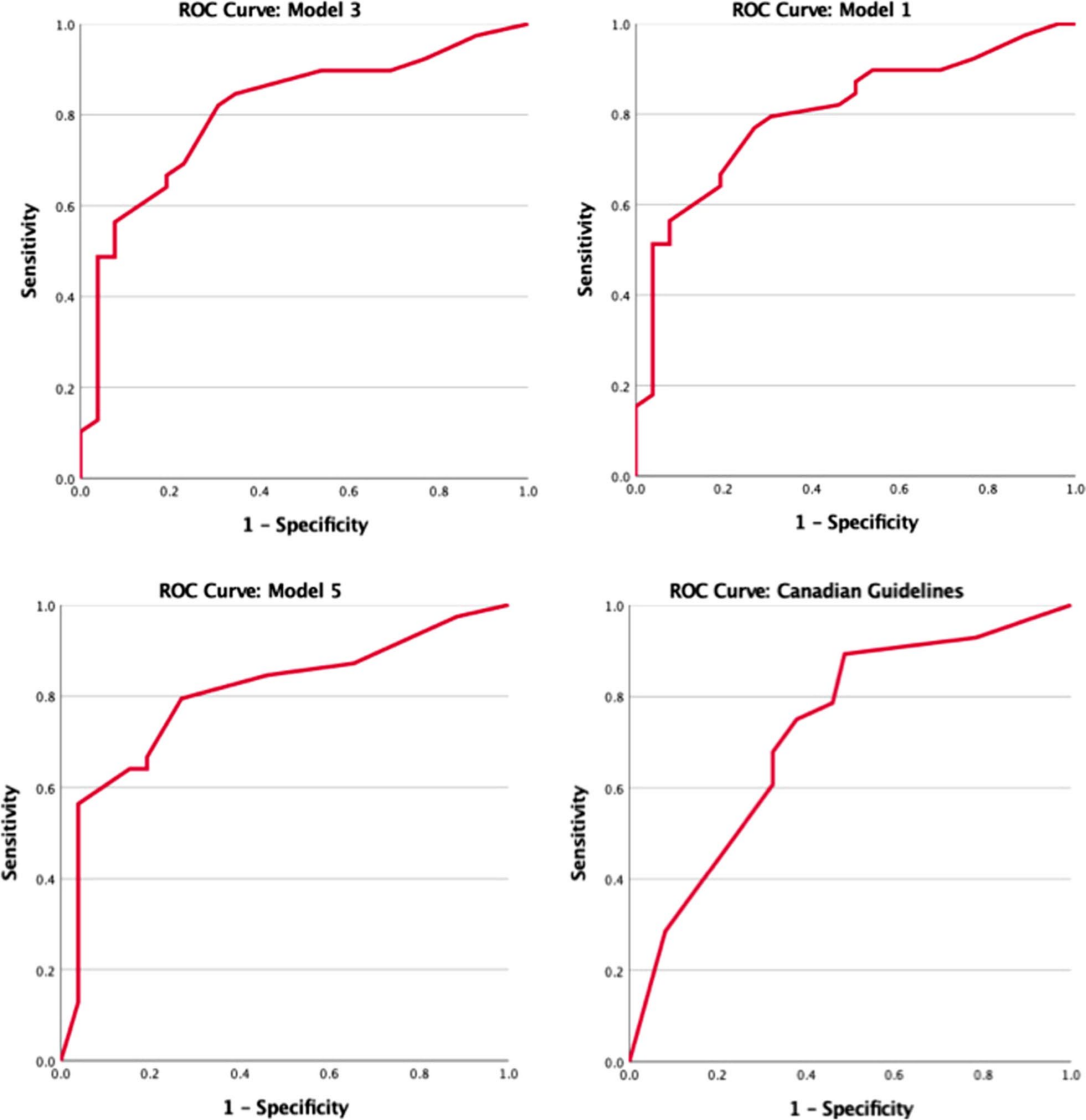
Top 3 model and Canadian Guideline model ROC curves

The variables chosen for tentative use in binary logistic regression passed all assumptions. This includes there being no independent variable that is highly correlated (r ≥ 0.70) with another independent variable used within the model (Table 2) [33]. Additionally, no independent variable demonstrated multicollinearity as all VIF values remained less than 5. Using the confirmed predictor variables; serum eosinophil, LM, LK, CRSwNP, asthma and SNOT-22, a total of 61 models were generated by the best subsets selection approach. A shortlist of 10 models were chosen based on higher Nagelkerke R^2^ scores which are listed in Table 3. The ROC curve AUC values for these 10 models were calculated and used to determine the top three models. In hierarchal order, the top 3 models were models 3, 1 and 5 with AUC of 0.808, 0.803 and 0.803 respectively (Table 3, Fig. 1). These scores for the Canadian Guideline for Biologic use in CRS can also be found in Tables 2 and 3 and depicted visually in Fig. 1.

**Table 2:** Pearson correlation coefficients for dichotomized biomarkers for type 2 Chronic Rhinosinusitis

	Pearson correlation coefficient (r)						
	Total IgE (ug/L)	Eosinophils (10*9/L)	LK score	SNOT-22	LM score	CRSwNP	Asthma
IgE	–	0.457*	0.178	− 0.164	0.224	0.252*	− 0.103
Eosinophils	0.457*	–	0.318*	− 0.145	0.335*	0.369*	− 0.140
LK score	0.178	0.318*	–	0.094	0.476*	0.566*	0.013
SNOT-22	− 0.164	− 0.145	0.094	–	0.198	0.162	− 0.025
LM	0.224	0.335*	0.476*	0.198	–	0.434*	− 0.149
CRSwNP	0.252*	0.369*	0.566*	0.162	0.434*	–	0.000
Asthma	− 0.103	− 0.140	0.013	− 0.025	− 0.149	0.000	–

CRSwNP: CRS with nasal polyps; Eos: Serum eosinophil; IgE: Serum Immunoglobulin E; LK: Lund Kennedy, LM: Lund Mackay; SNOT-22: Sino-nasal Outcome Test 22

**Table 3:** Nagelkerke R square, AUC scores and significance value for logistic regression models 1 to 10 and current Canadian Guidelines

Model	Variables Included	Nagelkerke R Square^a^	AUC	*p* value
Model 1	Asthma, CRSwNP, Eos, LK, LM, SNOT-22	0.355	0.803	0.000
Model 2	Eos, LK, LM, SNOT-22, CRSwNP	0.353	0.795	0.000
Model 3	Asthma, CRSwNP, Eos, LM, SNOT-22	0.353	0.808	0.000
Model 4	CRSwNP, Eos, LM, SNOT-22	0.350	0.796	0.000
Model 5	Asthma, CRSwNP, Eos, SNOT-22	0.343	0.803	0.000
Model 6	Asthma, CRSwNP, Eos, LK, SNOT-22	0.343	0.798	0.000
Model 7	Eos, SNOT-22, CRSwNP, LK	0.341	0.791	0.000
Model 8	Eos, SNOT-22, CRSwNP	0.340	0.795	0.000
Model 9	Asthma, Eos, LM, SNOT-22	0.339	0.799	0.000
Model 10	Asthma, Eos, LK, LM, SNOT-22	0.339	0.800	0.000
Canadian Guidelines	SNOT-22, CRSwNP, LK	0.145	0.691	0.009

^a^Closer to 1 the Nagelkerke R square is the better the goodness of fit

CRSwNP, CRS with nasal polyps; Eos, Serum eosinophilia; IgE, I ^18^mmunoglobulin E; LK, Lund Kennedy; LM, Lund Mackay; SNOT-22, Sino-nasal Outcome Test 22

Results from the binary logistic regression analysis for elevated serum IgE also provided insight into the performance of the top 3 models. Model 3 had the highest sensitivity (82.1%) and NPV (72%). Model 5 had the highest specificity (73.1%) and PPV (81.5%) (Table 4). Model 1 and 5 were tied for the lowest sensitivity (79.5%), while model 1 had the lowest specificity (69.2%), PPV (79.5%), and NPV (69.2%) (Table 4).

**Table 4:** Sensitivity, specificity, negative predictive value and positive predictive value for the top 3 models of elevated serum IgE

Order of Superiority	Model	Sensitivity	Specificity	PPV	NPV	AUC	*p* value
1	3	82.1	69.2	80.0	72.0	0.808	0.000
2	1	79.5	69.2	79.5	69.2	0.803	0.000
3	5	79.5	73.1	81.5	70.3	0.803	0.000
	Canadian Guidelines	87.2	34.2	66.7	64.3	0.691	0.009

PPV, Positive predictive value; NPV, Negative predictive value; AUC, area under the curve

*p value < 0.05 is statistically significant

Model 3 (variables: Asthma, CRSwNP, Eos, LM and SNOT-22) was determined to be the best model for predicting elevated serum IgE based on Nagelkerke R2 scores and AUC values. Amongst these 5 included variables only CRSwNP, Eosinophilia and Lund McKay increased the odds of elevated serum IgE with odds ratio values of 1.905, 7.477 and 2.038 respectively (Table 5). However, serum eosinophilia (*p* = 0.004) was the only variable to significantly contribute to the model.

**Table 5:** Significance value, odds ratio and confidence interval data for Model 3 variables

Variable	P2-value	OR	95% CI for OR	
			Lower	Upper
Asthma	0.667	0.711	0.235	2.523
CRSwNP	0.343	1.905	0.503	7.220
Eos	0.004	7.477	1.924	29.060
LM	0.427	2.038	0.352	11.808
SNOT-22	0.053	0.272	0.073	1.015

CI, Confidence interval; OR, Odds ratio

*p value < 0.05 is statistically significant

## Discussion

Biological treatments are indicated for uncontrolled severe type 2 CRSwNP [25–27]. The results of this study indicate that comorbid asthma, high serum eosinophil levels (≥ 300 cells/uL), CRSwNP phenotype, SNOT-22 ≥ 40 and LM scores ≥ 17 in combination provide an effective means of predicting IgE high type 2 inflammation. All five variables are intuitive markers for IgE high type 2 CRS for the following reasons. Variable one, asthma, has demonstrated a mutual type 2 inflammatory interaction with CRS when comparing upper and lower airway inflammatory endotypes [34, 35]. Variable 2, serum eosinophilia, has been shown to present concomitantly with elevated serum IgE levels in CRS [36, 37]. Together, eosinophils and IgE stimulate the production of type 2 inflammatory cytokines which results in polyp formation by suppressing tissue plasminogen activator and enabling fibrin crosslinking [38]. As expected, the next variable for type 2 inflammation was the end product—CRSwNP. Interestingly, multiple studies have identified type 2 inflammation in a large proportion of patients with CRSsNP, creating the possibility of expanding Canadian CRS biologic guidelines to include this population [20, 22–24]. However, the current study demonstrated that CRSsNP was not a suitable predictor for IgE high type 2 inflammation, which may reflect the geographical variability in CRSsNP endotypes [20, 21]. The subjective symptoms of an IgE high type 2 CRS can be accurately quantified as severe by SNOT-22 (variable 4) [18, 39]. Lastly, Hopkins 2017 and Rai 2019 indicated that variable 5—LM score—can be used to accurately assess polyp load, global sinus mucosal inflammation and CRS severity [40, 41].

**Fig. 2:**
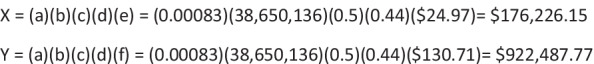
Calculations for estimating the yearly cost of IgE serology for rural Canadians. a = Incidence of CRSwNP per year: 0.00083 [43]; b = Proportion of patients with CRSwNP who experience disease recurrence after sinus surgery: 0.5 [26]; c = 2022 Canadian population: 38,650,136 [44]; d = Proportion of Canadians living in rural locations: 0.44 [17]; e = Low cost per sample: $24.97 [45, 46]; f = High cost per sample: $130.71 [45, 46]; X = Low cost of quantitative IgE serology per year; Y = High cost of quantitative IgE serology per year

Predicting IgE high type 2 CRSwNP using this biomarker model could optimize healthcare spending, improve healthcare equity and reduce social costs accrued by patients with CRS in rural and remote areas within Canada. According to phase 3 clinical trials (POLYP 1 and POLYP 2), patient weight (kg) and serum IgE levels are required to determine omalizumab dosing [14]. The IgE requirement should not be a barrier to treatment in tertiary centers as they tend to order more laboratory investigations than community hospitals [42]. However, this could be a barrier for community hospitals in rural and remote settings, where labs are limited to basic blood work and must send blood samples to the nearest tertiary centre for quantitative IgE serology. The estimated associated costs can range from $176,226.15 to $922,487.77 CAD per year for the greater than 7,000 rural Canadians who could be considered for omalizumab treatment (Fig. 2) [17, 18, 26, 43–46]. Through the use of this biomarker model, otolaryngologists in rural and remote settings can determine which patients have a high pretest probability for elevated serum IgE and are thus suitable for confirmatory IgE serology. This would improve healthcare resource allocation in the cost-intensive otolaryngology specialty, reduce the probability of laboratory error and make omalizumab more accessible [47, 48].

This algorithm could be seamlessly integrated into the care of patients with CRS who are recalcitrant to surgery and medical therapy as most variables are ordered preoperatively or during medical therapy [49]. If the eosinophil threshold for this algorithm was reduced to the mepolizumab threshold of > 150 cells/uL as per Ortega et al. 2014 an additional 13 patients would be considered ‘eosinophilic’ [50]. This reduced IgE model sensitivity to 76.9%, specificity to 42.3%, PPV to 66.7%, NPV to 55% and AUC to 0.714. Furthermore, utilizing eosinophilia thresholds specific to mepolizumab was shown to be less effective in predicting elevated IgE levels, which provides support for there being different patient populations suitable for mepolizumab and omalizumab. Therefore, with biomarker model 3 having a sensitivity of 82.1%, if the patient does not meet model 3 criteria they can both bypass IgE serology and be considered for dupilumab or mepolizumab treatment.

There could be additional benefits to this approach, however, more research is required to substantiate these assertions. One, understanding which patients have elevated serum IgE levels could indicate if they are to respond better or worse to omalizumab treatment. Phase 3 randomized studies–POLYP 1 and 2–prescribed omalizumab at doses that were based on weight (kg) and baseline IgE levels ranging from 72 to 3120 ng/mL. It is intuitive to believe that patients with elevated serum IgE are more likely to have a significant change in objective treatment outcomes due to the mechanism of action of omalizumab. Currently, there is one omalizumab study that demonstrated no significant impact of baseline serum IgE levels on the nasal polyp size and SNOT-22 score [51]. However, this study was limited by its small population (n = 23) which caused their regression analysis to not follow the one in ten rule [51]. Additional research is needed to confirm the impact of baseline IgE serology on omalizumab efficacy. Furthermore, the combination of study results and the need for IgE serology to dose omalizumab are likely to alter Canadian biologic guidelines to become more specific to each biologic type.

For instance, current Canadian biologic guidelines indicate that “patients with CRSwNP do not need another Type 2 inflammatory condition such as asthma to be considered for biologic therapy” [18]. This statement was supported by trials that focused on dupilumab, which at the time was the only biologic therapy approved by Health Canada for use in severe CRSwNP [52, 53]. Since this time, omalizumab has been approved [13]. Two randomized controlled trials demonstrated omalizumab’s efficacy in patients diagnosed with CRSwNP and comorbid severe asthma [54, 55]. However, POLYP 1 and 2 phase 3 trials indicated that mild to moderate asthma comorbidity was not a significant predictor of response to omalizumab treatment [14]. The current retrospective chart review did not include asthma severity in the binary logistic regression analysis, but the results support asthma diagnosis as an indicator for IgE high type 2 CRSwNP. This could also be integrated into future studies.

This study had limitations. First, age was recorded in this study but not included in statistical analysis because it was difficult to dichotomize this variable into high and low due to the stratiform pattern that arises when graphing type 2 inflammatory marker concentration to age in patients with type 2 CRSwNP [56]. This omission of age could have impacted results because type 2 cytokines tend to increase with age [56]. Allergen hypersensitivity status also could not be included as a confounder covariate due to the sample size preventing additional variables from being studied as per the one in ten rule [31]. Patient cytokine profiles also vary by geographic location, thus by only including patients from British Columbia the generalizability of this study’s findings could be reduced [21]. Future studies should be encouraged to recruit a larger sample population, randomly select patients and involve multiple centres to allow for allergen hypersensitivity and geography to be included as study variables.

## Conclusion

CRS is a chronic and debilitating inflammatory disease that can greatly reduce a patient’s quality of life. IgE high type 2 CRS can be accurately predicted by a biomarker model consisting of eosinophils ≥ 300 cell/uL, CRSwNP, LM ≥ 17, comorbid asthma and SNOT-22 ≥ 40. Utilizing this model to triage IgE serology could improve resource allocation and reduce social costs. Additional high-quality research is required to determine if baseline serum IgE levels impact the efficacy of omalizumab.

## Data Availability

The datasets used and/or analyzed during the current study are available from the corresponding author on reasonable request.

## Abbreviations

CRS: Chronic rhinosinusitis
CRSwNP: Chronic rhinosinusitis with nasal polyps
CRSsNP: Chronic rhinosinusitis without nasal polyps
IL: Interleukin
Ig: Immunoglobulin
UBC: University of British Columbia
REDCap: Research electronic data capture
FESS: Functional endoscopic sinus surgery
LM: Lund-MacKay
LK: Lund Kennedy
SNOT-22: Sino-nasal outcome test-22
VIF: Variance inflation factor
EPOS: European position paper on rhinosinusitis and nasal polyps
EUFOREA: European forum for research and education
ROC: Receiver operator characteristic
AUC: Area under the curve
PPV: Positive predictive value
NPV: Negative predictive value

## References

[1] Chen Y, Dales R, Lin M (2003). The epidemiology of chronic rhinosinusitis in canadians. Laryngoscope.

[2] DeConde AS, Soler ZM (2016). Chronic rhinosinusitis: epidemiology and burden of disease. Am J Rhinol Allergy.

[3] Tomoum MO, Klattcromwell C, DelSignore A, Ebert C, Senior BA (2015). Depression and anxiety in chronic rhinosinusitis: depression/anxiety in chronic rhinosinusitis. Int Forum Allergy Rhinol.

[4] Kaplan A (2013). Canadian guidelines for chronic rhinosinusitis: clinical summary. Cam Fam Physician.

[5] Alt JA, Thomas AJ, Curtin K, Wong J, Rudmik L, Orlandi RR (2017). Mortality risk in patients with chronic rhinosinusitis and its association to asthma: Chronic rhinosinusitis and mortality. Int Forum Allergy Rhinol.

[6] Husain Q, Sedaghat AR (2019). Understanding and clinical relevance of chronic rhinosinusitis endotypes. Clin Otolaryngol.

[7] Kuruvilla ME, Lee FEH, Lee GB (2019). Understanding asthma phenotypes, endotypes, and mechanisms of disease. Clinic Rev Allerg Immunol.

[8] Smith M. Dupixent now approved by Health Canada for patients with severe asthma. Sanofi. 2020. http://sanoficanada.mediaroom.com/2020-11-17-DUPIXENT-R-dupilumab-injection-now-approved-by-Health-Canada-for-patients-with-severe-asthma. Accessed July 2021.

[9] Product monograph, including patient medication information: Dupixent (Duplimab Injection). Sanofi-aventis Canada Inc.; 2020:1–70.

[10] Hellings PW, Verhoeven E, Fokkens WJ (2021). State-of-the-art overview on biological treatment for CRSwNP. Rhin.

[11] Karp J, Dhillon I, Panchmatia R, Javer A (2021). Subcutaneous mepolizumab injection: an adjunctive treatment for recalcitrant allergic fungal rhinosinusitis patients with asthma. Am J Rhinol Allergy.

[12] Kaplan AP, Giménez-Arnau AM, Saini SS (2017). Mechanisms of action that contribute to efficacy of omalizumab in chronic spontaneous urticaria. Allergy.

[13] Regulatory decision summary - Xolair - Health Canada. Government of Canada. 2021. https://hpr-rps.hres.ca/reg-content/regulatory-decision-summary-detail.php?lang=en&linkID=RDS00839. Accessed July 2021.

[14] Gevaert P, Omachi TA, Corren J (2020). Efficacy and safety of omalizumab in nasal polyposis: 2 randomized phase 3 trials. J Allergy Clin Immunol.

[15] Agache I, Song Y, Alonso-Coello P (2021). Efficacy and safety of treatment with biologicals for severe chronic rhinosinusitis with nasal polyps: a systematic review for the EAACI guidelines. Allergy.

[16] CADTH Reimbursement Review Dupilumab (Dupixent). Canadian Journal of Health Technologies. 2021. https://cadth.ca/sites/default/files/cdr/complete/SR0667-combined%20clinical%20and%20PE%20report.pdf

[17] Population size and growth in Canada: Key results from the 2016 Census. Statistics Canada. 2016. https://www150.statcan.gc.ca/n1/daily-quotidien/170208/dq170208a-eng.html. Accessed Dec 2021.

[18] Thamboo A, Kilty S, Witterick I (2021). Canadian Rhinology Working Group consensus statement: biologic therapies for chronic rhinosinusitis. J of Otolaryngol Head Neck Surg.

[19] Harris P (2018). Research electronic data capture (REDCap). J Med Libr Assoc.

[20] Stevens WW, Peters AT, Tan BK, et al. Associations between inflammatory endotypes and clinical presentations in chronic rhinosinusitis. J Allergy Clin Immunol. 2019;7(8):2812–2820.e3. 10.1016/j.jaip.2019.05.009 PMC6842686 31128376

[21] Wang X, Zhang N, Bo M (2016). Diversity of T H cytokine profiles in patients with chronic rhinosinusitis: a multicenter study in Europe, Asia, and Oceania. J Allergy Clin Immunol.

[22] Tan BK, Klingler AI, Poposki JA (2017). Heterogeneous inflammatory patterns in chronic rhinosinusitis without nasal polyps in Chicago, Illinois. J Allergy Clin Immunol.

[23] Tyler MA, Russell CB, Smith DE (2017). Large-scale gene expression profiling reveals distinct type 2 inflammatory patterns in chronic rhinosinusitis subtypes. J Allergy Clin Immunol.

[24] Delemarre T, Holtappels G, De Ruyck N (2020). Type 2 inflammation in chronic rhinosinusitis without nasal polyps: another relevant endotype. J Allergy Clin Immunol.

[25] Fokkens WJ, Lund VJ, Hopkins C (2020). European position paper on rhinosinusitis and nasal polyps. Rhinology.

[26] Fokkens WJ, Lund V, Bachert C (2019). EUFOREA consensus on biologics for CRSwNP with or without asthma. Allergy.

[27] Bachert C, Han JK, Wagenmann M (2021). EUFOREA expert board meeting on uncontrolled severe chronic rhinosinusitis with nasal polyps (CRSwNP) and biologics: definitions and management. J Allergy Clin Immunol.

[28] Mustafa SS, Vadamalai K, Scott B, Ramsey A (2021). Dupilumab as add-on therapy for chronic rhinosinusitis with nasal polyposis in aspirin exacerbated respiratory disease. Am J Rhino l Allergy.

[29] Lombardo N, Pelaia C, Ciriolo M (2020). Real-life effects of benralizumab on allergic chronic rhinosinusitis and nasal polyposis associated with severe asthma. Int J Immunopathol Pharmacol.

[30] Wuensch K. Binary Logistic Regression with SPSS. East Caroline University Department of Psychology. http://core.ecu.edu/psyc/wuenschk/MV/multReg/Logistic-SPSS.pdf

[31] Peduzzi P, Concato J, Kemper E, Holford TR, Feinstein AR (1996). A simulation study of the number of events per variable in logistic regression analysis. J Clin Epidemiol.

[32] Min JY, Tan BK (2015). Risk factors for chronic rhinosinusitis. Curr Opin Allergy Clin Immunol.

[33] Asuero AG, Sayago A, González AG (2006). The correlation coefficient: an overview. Crit Rev Anal Chem.

[34] Kariyawasam HH, James LK (2020). Chronic Rhinosinusitis with nasal polyps: targeting IgE with anti-IgE Omalizumab therapy. DDDT.

[35] Kanemitsu Y, Suzuki M, Fukumitsu K (2020). A novel pathophysiologic link between upper and lower airways in patients with chronic rhinosinusitis: association of sputum periostin levels with upper airway inflammation and olfactory function. World Allergy Organ J.

[36] Cao PP, Zhang YN, Liao B (2014). Increased local IgE production induced by common aeroallergens and phenotypic alteration of mast cells in Chinese eosinophilic, but not non-eosinophilic, chronic rhinosinusitis with nasal polyps. Clin Exp Allergy.

[37] Wei B, Liu F, Zhang J (2018). Multivariate analysis of inflammatory endotypes in recurrent nasal polyposis in a Chinese population. Rhin.

[38] Schleimer RP (2017). Immunopathogenesis of chronic rhinosinusitis and nasal polyposis. Annu Rev Pathol.

[39] Khan AH, Reaney M, Guillemin I, et al. (2021) Development of Sinonasal Outcome Test (SNOT ‐22) domains in chronic rhinosinusitis with nasal polyps. Laryngoscope. 10.1002/lary.29766 PMC9292332 34437720

[40] Rai G, Roy P, Gupta N (2019). Computed tomography score an excellent marker: differentiates eosinophilic and non-eosinophilic variants of chronic rhinosinusitis with nasal polyps. Indian J Otolaryngol Head Neck Surg.

[41] Hopkins C, Browne JP, Slack R, Lund V, Brown P (2007). The Lund-Mackay staging system for chronic rhinosinusitis: how is it used and what does it predict?. Otolaryngol Head Neck Surg.

[42] Valencia V, Arora VM, Ranji SR, Meza C, Moriates C (2018). A comparison of laboratory testing in teaching vs nonteaching hospitals for 2 common medical conditions. JAMA Intern Med.

[43] Tan BK, Chandra RK, Pollak J (2013). Incidence and associated premorbid diagnoses of patients with chronic rhinosinusitis. J Allergy Clin Immunol.

[44] Canada’s population clock (real-time model). Statistics Canada. https://www150.statcan.gc.ca/n1/pub/71-607-x/71-607-x2018005-eng.htm

[45] McClernon A, Freeman A, Cheeley R, McClernon D. Cost Comparison of Shipping Frozen Plasma vs. Ambient Temperature using vivest. Vivebio.com. https://vivebio.com/wp-content/uploads/2017/09/2012-12-09-dart-cost-comparison-of-shipping-frozen-plasma-versus-ambient-temperature-using-vivest.pdf

[46] Schedule of fees for laboratory services outpatient. British Columbia Ministry of Health. Published June 1, 2020. http://www.bccss.org/bcaplm-site/Documents/Programs/laboratory_services_schedule_of_fees.pdf. Accessed July 2021.

[47] Restelli V, Taylor A, Cochrane D, Noble MA (2017). Medical laboratory associated errors: the 33-month experience of an on-line volunteer Canadian province wide error reporting system. Diagnosis.

[48] Thomas AJ, McCoul ED, Meier JD, Newberry CI, Smith TL, Alt JA (2020). Cost and operative time estimation itemized by component procedures of endoscopic sinus surgery. Int Forum Allergy Rhinol.

[49] Rosenfeld RM, Piccirillo JF, Chandrasekhar SS (2015). Clinical practice guideline (update): adult sinusitis. Otolaryngol Head Neck Surg.

[50] Ortega HG, Liu MC, Pavord ID (2014). Mepolizumab treatment in patients with severe eosinophilic asthma. N Engl J Med.

[51] Armengot-Carceller M, Gómez-Gómez MJ, García-Navalón C (2021). Effects of omalizumab treatment in patients with recalcitrant nasal polyposis and mild asthma: a multicenter retrospective study. Am J Rhinol Allergy.

[52] Gevaert P, Langloidolt D, Lackner A (2006). Nasal IL-5 levels determine the response to anti–IL-5 treatment in patients with nasal polyps. J Allergy Clin Immunol.

[53] Bachert C, Mannent L, Naclerio RM (2016). Effect of subcutaneous Dupilumab on nasal polyp burden in patients with chronic sinusitis and nasal polyposis: a randomized clinical trial. JAMA.

[54] Gevaert P, Calus L, Van Zele T (2013). Omalizumab is effective in allergic and nonallergic patients with nasal polyps and asthma. J Allergy Clin Immunol.

[55] Pinto JM, Mehta N, DiTineo M, Wang J, Baroody FM, Naclerio RM (2010). A randomized, double-blind, placebo-controlled trial of anti-IgE for chronic rhinosinusitis. Rhin.

[56] Ryu G, Dhong H, Park M (2020). Age-associated changes in chronic rhinosinusitis endotypes. Clin Exp Allergy.

